# A Critical Review on the Effect of Docosahexaenoic Acid (DHA) on Cancer Cell Cycle Progression

**DOI:** 10.3390/ijms18081784

**Published:** 2017-08-17

**Authors:** Marnie Newell, Kristi Baker, Lynne M. Postovit, Catherine J. Field

**Affiliations:** 1 Department of Agricultural Food and Nutritional Science, Faculty of Agricultural, Life and Environmental Sciences, 4-126 Li Ka Shing Center for Health Research Innovation, University of Alberta, Edmonton, AB T6G 2E1, Canada; marnie.newell@ualberta.ca; 2 Department of Oncology, Faculty of Medicine and Dentistry, University of Alberta, Edmonton, AB T6G 2E1, Canada; kbaker2@ualberta.ca (K.B.); postovit@ualberta.ca (L.M.P.)

**Keywords:** cancer, cell cycle, cyclins, docosahexaenoic acid (DHA), G1 phase, G2M phase, omega-3, synergistic, proliferation, arrest, chemotherapy

## Abstract

Globally, there were 14.1 million new cancer diagnoses and 8.2 million cancer deaths in 2012. For many cancers, conventional therapies are limited in their successes and an improved understanding of disease progression is needed in conjunction with exploration of alternative therapies. The long chain polyunsaturated fatty acid, docosahexaenoic acid (DHA), has been shown to enhance many cellular responses that reduce cancer cell viability and decrease proliferation both in vitro and in vivo. A small number of studies suggest that DHA improves chemotherapy outcomes in cancer patients. It is readily incorporated into cancer cell membranes and, as a result there has been considerable research regarding cell membrane initiated events. For example, DHA has been shown to mediate the induction of apoptosis/reduction of proliferation in vitro and in vivo. However, there is limited research into the effect of DHA on cell cycle regulation in cancer cells and the mechanism(s) by which DHA acts are not fully understood. The purpose of the current review is to provide a critical examination of the literature investigating the ability of DHA to stall progression during different cell cycle phases in cancer cells, as well as the consequences that these changes may have on tumour growth, independently and in conjunction with chemotherapy.

## 1. Introduction

**Figure 1:**
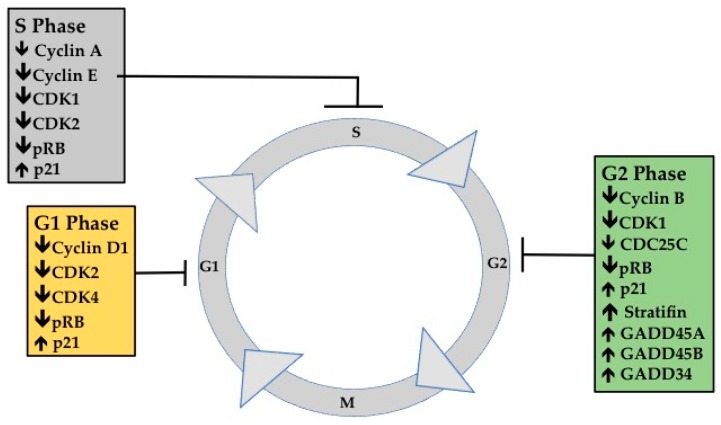
Schematic illustrating the pleiotropic effects of docosahexaenoic acid (DHA) on genes and proteins throughout the cell cycle in various cancer cell types (referenced in accompanying Table 1).

Cancer is one of the most frequently diagnosed diseases worldwide, accounting for an estimated 14.1 million new diagnoses in 2012. It is the second most frequent cause of death and, in 2015 it accounted for 8.8 million deaths. Of those, lung (1.69 million), liver (788,000), colorectal (774,000), stomach (754,000) and breast (571,000) cancers were the most common causes of mortality [1]. These statistics highlight the limited success of conventional therapies, possibly due to increased drug resistance of tumours or targeted therapies being ineffective against heterogeneous tumours. It is clear that an improved understanding of disease progression is needed in conjunction with exploration of alternative therapies. Epidemiological studies have established a link between fatty fish and reduced incidence of certain cancers-specifically colorectal, prostate and breast [2]. It is well known that one of the key bioactive components in fatty fish is the long chain polyunsaturated fatty acid (LCPUFA) docosahexaenoic acid (DHA). Providing DHA has been shown to enhance many cellular responses including anti-inflammatory actions, modulated oxidative stress response and activation of peroxisome proliferator-activated receptors (PPARs) [3] and it has many potential mechanisms of action in cancer [2,4]. It is readily incorporated into cancer cell membranes, primarily at the sn-2 position (commonly with a saturated fatty acid like palmitic and stearic acid in the sn-1 position) and preferentially esterified to phosphatidylethanolamine (PE), found in the inner leaflet of the plasma membrane, with lesser amounts in phosphatidylcholine (PC) (outer leaflet of the plasma membrane), and the other phospholipids (PLs) [5]. As a result, there has been considerable research aimed at identifying the role of DHA in cell membrane initiated events. The capacity of DHA to induce cancer cell apoptosis or reduce proliferation in vitro and in vivo through potential mechanisms including membrane incorporation, lipid peroxidation [6,7], eicosanoid metabolites or action on nuclear receptors, is well-documented (reviewed by D’Eliseo and Velotti [8]), yet there is limited research into the effects of DHA on cell cycle regulation in cancer cells. The aim of this review is to provide a critical examination of studies investigating the ability of DHA to stall progression during different cell cycle phases (Section 2 and Section 3; Table 1 and Table 2 and Figure 1) in cancer cells. This functionality of DHA in cancer could lead to growth inhibition, independently and in conjunction with chemotherapy.

## 2. Cell Cycle Progression in Cancer

Progression through the cell cycle is tightly regulated and checkpoints at phase transitions during the cell cycle ensure that only healthy cells progress and proliferate. Loss of cell cycle control is one of the hallmarks of cancer [30]. Normal, non-cancerous cells monitor their environments and have the potential to either remain quiescent, proliferate, or become post-mitotic [30]. Once a cell is committed to entering the cell cycle, checkpoints regulated by cyclins and their associated cyclin dependent kinases are in place to monitor errors and avoid mutations [31]. In a cancer cell with dysregulated growth, these genes and proteins are frequently overexpressed and checkpoint control is evaded. The key cell cycle checkpoint between G_2_ and M phase was investigated in a retrospective cohort of breast tumour samples from 10 ER^+^(and/or)PR^+^/HER2^+^; 32 ER^+^(and/or)PR^+^/HER2^−^; 1 ER^−^PR^−^/HER2^+^ and 4 ER^−^PR^−^/HER2^−^ patients. Seventy-six mitotic checkpoint genes were analyzed by RTPCR. It was found that *NDC80*, *BUB1*, *BUB1B*, *CCNB1*, *TACC3*, *TPX2*, *CCNA2*, *CDC2* and *CDC20* were significantly up-regulated in all tumour types compared to normal breast tissue and in addition to these genes, while *NEK2*, *CENPE*, *BIRC5*, *CCNB2*, *AURKB*, *AURKA*, *TTK* and *PLK1* were found to be highly up-regulated in invasive breast tissues compared to normal breast tissues [32]. Some of these same mitotic genes are being investigated as emerging cancer therapy targets including ones (i.e., *AURKA* and *PLK1*) that are implicated in cancers with “poorer prognosis”. To date, the thirty AURK inhibitors tested have performed poorly in clinical trials, while PLK1 inhibitors have shown potential in solid tumours [33]. However, these results have not yet been reproduced in a clinical setting. Although many emerging cell cycle targets are still being investigated, currently there is no highly effective anti-mitotic drug that works in solid tumours and in patients [34].

## 3. Effect of Docosahexaenoic Acid on Cell Cycle Progression

DHA has been shown to be cytotoxic to many cancer cell types and to have differential effects on a broad variety of cellular molecules and pathways; the mechanisms proposed to explain this may be phenotype-specific although this has not been clearly established [8]. Furthermore, the ability of DHA to alter cancer cell progression through the cell cycle has not been extensively investigated. Cell cycle analysis was assessed in all studies by propidium iodide staining of cells and flow cytometric analysis. Of 21 studies, nine reported cell cycle stall at G1, three reported alterations in S phase, five reported cell cycle stall in G2M and another four studies reported that multi-phases were affected. Figure 1 and Table 1 provide a summary of cell cycle markers that have been reported to change with DHA treatment.

### 3.1. G1 Phase Analysis

Treatment of cells with DHA stalled cell cycle progression in the G1 phase of the cell cycle in leukemia [12], colorectal [14], neuroblastoma [13] and breast [15,17,18] cancer cells. Acute myeloid leukemia cells (KG1A) treated with 100 μM DHA were analyzed at 0, 2, 4, 6, and 24 h for cell cycle progression. At T = 0, G1 = 57%, S = 31% and G2M 11% for both control and DHA-treated cells. Although there was a slight increase in G1 = 60%, no other changes were observed after the first 4 h, after 24 h the control and DHA-treated cells in the G1 phase were, 56% and 68% respectively. In comparison, control cells had 56% in G1 after 24 h. Although the trend did not reach statistical significance, the higher proportion of DHA-treated cells stalled in G1 suggests the propensity for DHA treatments to induce cell cycle arrest. A similar trend was also observed in HL-60 leukemia cells where a 12–22% increase in G1 arrest was seen [35]. There were no changes in S phase and the proportion of cells in G2M decreased, but not significantly [12]. Cell cycle analysis of breast [15] and neuroblastoma [13] cells showed a concentration-dependent (25–150 μM in breast; 0–70 μM in neuroblastoma) increase in percentage of cells stalled at G1. However, in both studies, a concentration of 25 μM was sufficient to induce a significant effect. In LA-N-1 neuroblastoma cells, it was also found that DHA treatment had a marginally increased yet non-significant efficacy over eicosapentaenoic acid (EPA) treatments at the same concentration [13]. Xue et al. compared human breast cells and mouse breast cells and also found a higher percentage of cells in G1 phase following a treatment with increasing concentrations of DHA in both cell types and a corresponding decrease in the S and G2M phases [15]. A separate study looked at FM3A mouse breast cancer cells and found a significant increase in the proportion of cells in G1 and a corresponding decrease in S phase with 10 μM treatment for 12 h [17]. In another study, HT-29 colorectal cells were serum starved to synchronize their cell cycle to G1 and then stimulated with 150 μM DHA treatment. This resulted in an increase from 32% to 63% in the G1 phase of the cell cycle; a decrease from 47% to 30% in S phase; and a reduction in G2 from 21% to 8% [14]. Rescigno et al. found that MCF10A (non-tumorigenic breast cells) and SKBR3 breast cancer cells treated with DHA (100 or 300 μM) had a greater proportion of cells in G1 after 24, 48 and 72 h, while MCF-7 cells had a lower proportion of cells in G1 compared to control at the 48 and 72 h timepoints [18].

In conclusion, a consistent propensity to stall during the G1 phase when treated with DHA was seen across four different cancer types with varied experimental conditions. In four of these studies (three on breast [15,16,18] and one on leukemia [12]), cells were not synchronized prior to the flow cytometry experiments. Synchronization of the cell cycle by serum starvation moves the cells to the G1 phase before replacing the serum and starting treatments. This limitation was overcome by other strengths in the studies: all three breast cancer cell studies assessed multiple cell lines and different DHA concentrations [15,16,18]. Yamagami et al. assessed time point variation so an increasing trend of cells accumulating in G1 could be seen over time [12]. These studies used varying concentrations of DHA from 10 to 300 μM. It has been reported that, in serum-starved cells, the concentration of DHA required to elicit a response can be four times lower than that required in the presence of serum [20]. For example, in the experiment where breast cells were treated with 10 μM, they were grown with only 0.2% serum providing a possible explanation for the efficacy of DHA at this concentration [17]. Only one study investigating DHA treatment in conjunction with chemotherapy reported cell cycle arrest in the G1 phase. In this study, gastric AGS cells were treated with 30 μg/mL DHA ± 12.5 μg/mL 5-fluorouracil (5-FU) for 48 h. The percentage of cells in the G1 phase was significantly increased compared to control following both DHA (19% higher), and 5-FU (33% higher) treatments; but no significant differences were observed between the two treatments. The effect of the DHA + 5-FU combination was significantly higher with respect to individual treatments (39%). The percentage of cells in S-phase decreased with treatment: 56% in the Control group, 34% in the DHA group, 25% in the 5-FU group, and 19% in the DHA + 5-FU group. This study also assessed the effect of DHA + 5-FU on the expression of mitochondrial electron transfer chain (METC) complexes and at 48 h DHA + 5-FU decreased significantly their expression, differently from control and individual treatments. These results suggest that DHA and/or 5-FU inhibit entry/exit to the METC, therefore disrupting energy metabolism within the cell [19]. Interestingly, two of the studies (focused on lung [36] and neuroblastoma [13]) that reported G1 arrest with DHA and in the absence of chemotherapy, also documented a decrease in mitochondrial membrane potential suggesting an effect of DHA on the mitochondrial function in a cancer cell.

### 3.2. G1 Phase Cellular Markers

Progression through G1 is regulated by Cyclin-dependent kinases (CDK) 2, 4 and 6; Cyclins D1, -2, -3; and Cyclin E [13], and it has been suggested that G1/S phase arrest is due in part to increased levels of p21, p27 and p53 and decreased levels of Cyclin D1 [37]. p21 and p27 are CDK inhibitors that inhibit Cyclin D/CDK4/6 and Cyclin E/CDK2 complexes [29]. Conflicting results have been reported for the effects of DHA on Cyclin D1. After DHA treatment, expression of Cyclin D1 was found to be reduced in lung [36], MCF-7, 4T1 breast [15] and KLP-1 breast [30] cells while other studies found no changes in Cyclin D1 expression in FM3A [17], MDA-MB-231, MCF-7 or SK-BR-3 breast cells [16]. Taken together, these results highlight the heterogenicity among different immortalized cell lines, not only in baseline differences in expression of cell cycle proteins but also in the response of cell cycle genes to DHA treatment. In HT-29 colorectal cells, treatment with DHA but not EPA, linoleic acid (LA), α linolenic acid (ALA), nor arachidonic acid (AA) reduced activation of Cyclin D1, E and A-dependent histone 1 kinases. The authors suggest that DHA treatment resulting in reduced Cyclin A protein expression (confirmed by a Western blot) could be responsible for the effects seen on Cyclin A-dependent histone 1 kinase. Cyclin D1 or E protein expression was not measured [14]. In a separate experiment, the antioxidant BHT was added with DHA and resulted in a reversal of the effects on Cyclin A. The authors suggest that cell cycle arrest could be due in part to increased oxidative stress with DHA treatment [14]. It should be noted that, while there is considerable evidence that treatment with DHA increases the amount of cytotoxic lipid peroxidation products [6,7] with subsequent induction of apoptosis [8], this study [14] was one of only two to link lipid peroxidation with cell cycle arrest. Decreased CDK2 protein expression was found in lung [38] and neuroblastoma cells [13]; in fact, CDK2 relative expression of cells treated with 50 μM DHA was approximately 50% less than without DHA treatment [13]. Similarly, Cyclin E expression was reduced to 30% of control with 50 μM DHA in these cells [13]. FM3A mouse breast cells also showed reduced Cyclin E and pCDK2 expression after DHA treatment [17].

Regulation of cell cycle at the molecular level is maintained by the retinoblastoma protein (pRb), which sequesters E2F when in a hypophosphorylated state, and thereby inhibits proliferation [9]. Hypophosphorylated pRB was reported in lung [38], colorectal [14] and mouse breast cancer [17] cells after DHA treatment. In both the lung and colorectal studies pRb levels in DHA-treated cells were compared to oleic acid or linoleic fatty acid-treated cells [14,38]. Under normal circumstances, the phosphorylation of pRB results in its dissociation and release of E2F-1 which then induces entry to S-phase [17]. Reduced activity of E2F-1 measured by a gel shift assay was seen in DHA-treated colorectal cells [14]. In an in vivo model, Jiang et al. induced mammary tumours in rats and in addition to reduced tumours in rats fed a high *n*-3 diet, they also found lower Cyclin D1, pRB and higher p21 and p27 protein expression following DHA treatment [32]. Levels of p53 and p21 increased in a time-dependent manner in KLP-1 [30], MCF-10A, mCF-7 and SK-BR-3 [18] breast cells treated with DHA. Although none of the studies to date have provided a complete analysis of all cell cycle markers, there appears to be consensus on a reduction of Cyclin D [14,15], CDK2 [13], and pRb [14,38]. The expression of p21 was found to be decreased in MCF-7 [16,18] and SK-BR-3 [16] breast cancer cells but was not changed in MDA-MB-231 breast cancer cells [16]. p27 protein expression was not changed in MDA-MB-231, MCF-7 or SK-BR-3 cells [16], but was increased in mouse breast cells, although the mRNA expression was unchanged [17], suggesting a post-transcriptional effect of DHA on this protein. It is known that ERK1/2 and STAT3 phosphorylation leads to an increase in cell proliferation and survival as STAT3 up-regulates Cyclin D1 and p21 expression [39]. In SKBR3, and to lesser extent in MCF-7 cells, there was decreased phosphorylation of ERK1/2 and STAT3 after DHA treatment [18], suggestive of a mechanism involving reduced STAT3 activation of p21 leading to G1 arrest. Together, these studies provide evidence that DHA treatment can stall cancer cells in G1 due in part to decreased Cyclin D, possibly via the associated kinase CDK2 and can prevent pRb phosphorylation and E2F-1 activation.

### 3.3. S Phase Analysis

Only three studies were found reporting that DHA treatment affects transition through the S phase of the cell cycle. In the hepatocarcinoma cell line MHCC97L, 50 μM DHA was found to disrupt and prolong S phase transition [22]. In this experiment, cells were BrdU-labelled and DNA synthesis time, based on the relative movement of cells through the cell cycle, was measured. The time to progress through S phase increased from 18 to 21 h after DHA treatment. In particular, the proportion of Jurkat leukemia cells in the S phase of the cell cycle (measured at 0 and 24 h) increased from 30% to 68% with 30 μM DHA treatment while G1 decreased (62% to 43%) [22]. Control cells had a larger proportion of cells moving to G2 than the DHA treatment (11% vs. 2%), suggesting that cells were continuing to move through the cell cycle compared to the DHA cells that were accumulating in S phase [20]. Albino et al. compared two melanoma lines, one that was sensitive (SK-Mel-110) and one that was refractory (SK-Mel-29) to DHA and found that the SK-Mel-110 cells accumulated in the S-phase (36% compared to 17%) with 2 μg/mL DHA but no changes were seen in SK-Mel-29 with DHA treatment [21]. The serum starvation, which induces cellular stress, in the leukemia and melanoma cells could explain the effectiveness of low concentrations of DHA used in these studies.

### 3.4. S Phase Cellular Markers

Cyclin E controls entry from late G1 to S phase and this is followed, as DNA synthesis begins, by an increase in Cyclin A and associated CDK2 Cyclin A. CDK2 protein expression was found to be lower in metastatic MHCC97L liver cells [22] and leukemia [20] cells treated with DHA. DHA also decreased Cyclin E protein expression and decreased COX-2 mRNA expression in MHCC97L cells [22]. COX-2 is known to be overexpressed in many cancer types resulting in inhibition of apoptosis [8] and the authors suggest that further studies should be conducted in order to determine the relationship between reduced COX-2 and cell cycle arrest [22]. Although the proliferation marker pRb was hypophosphorylated in leukemia [20] cells and SK-Mel-110 melanoma cells. No other cell markers (Cyclin D, Cyclin E, p21 or p27) were different in this melanoma cell line after DHA treatment [21]. Siddiqui et al. reported that a treatment with 10 μM DHA resulted in membrane incorporation, sphingomyelinase activation and a four-fold increase in ceramide generation [20]. The authors proposed a pathway for DHA-mediated cell cycle arrest in S-phase in leukemia cells: initiation of p21 activation, which in turn leads to inhibition of the CDK2/Cyclin A complex, hypophosphorylation of pRb and subsequent S-phase arrest [20].

### 3.5. G2M Phase Analysis

The G2M checkpoint is a known target for cell cycle inhibition [34] and the ability for DHA to arrest cells at this point has been studied in leukemia [25], pancreatic [23], breast [18,24,30], and colorectal [28,29] cancer cells. In a study comparing treatments with *n*-3 (mixture of EPA and DHA) or *n*-6 fatty acid emulsion (Omegaven), pancreatic cells (MIA PaCa-2) were found after 24 h to accumulate in the G2M phase only when treated with 100 μM *n*-3 emulsion [23]. However, at 48 h there was only a small further increase suggesting that by 48 h cells had moved on to cell death/apoptosis [23]. In a synchronized MDA-MB-231 breast cancer cell population, treatment with increasing concentrations of DHA resulted in cells stalled at 18 h in the G2M phase of cell cycle in a dose-dependent manner [24]. In the study by Rescigno et al., a higher proportion of SKBR3 cells were found in G2M with 100 and 300 μM treatment compared to control at 24, 48 and 72 h [18]. There is a growing number of studies demonstrating the synergistic efficacy of DHA in conjunction with chemotherapy (reviewed by D’Eliseo and Velotti [8]), however only three studies have reported cell cycle analysis. In CaCo_2_ cells treated with a (0.36 mL/L) fish oil (FO) emulsion it was found that there was a 2.2-fold increase in G2M with FO alone, but in combination with 5-FU, cells increased in the S phase from ~40% to 70% [27]. This study did not look at any cell cycle marker to confirm the flow cytometric analysis of cell cycle and the FO was a mixture of EPA and DHA, so some of these effects could be attributed to EPA, which has been proposed to have a different mechanism to explain anti-cancer effects on tumour cells [27]. In a panel of three different leukemia cell lines and three different antineoplastic drugs, it was found that treatment with 50 μM DHA elicited G2M arrest in JVM-2 and MEC-2 cells but not in EHEB cells. The addition of 1.5 μM doxorubicin or 42 μM fludarabine to 75 μM DHA elicited G2M arrest in EHEB cells. JVM-2 and MEC-2 leukemia cells treated with 50 μM DHA + 1.5 μM doxorubicin treatment or 100 nM of vincristine showed an increase in G2M arrest compared to DHA treatment alone. EPA and LA were also tested and LA did not induce cell cycle arrest and, although EPA elicited a response, it was less efficacious compared to DHA confirming, at the same dose, a differential ability of *n*-3 fatty acids to slow growth of malignant B-lymphocytes. The combination of DHA + doxorubicin (in EHEB, JVM-2 and MEC-2) or DHA + vincristine (in JVM-2 and MEC-2) showed increased chemo-sensitivity over chemotherapy alone highlighting the synergistic effects of DHA and chemotherapy [25]. In prostate LNCaP and PC3 cells, an enhanced effect was found with a dose of 25 μM DHA and 0.6 nM docetaxel (TXT). The synergism was found to be best at 48 h and by 72 h the beneficial results began to diminish. This could possibly be due to a limitation of available DHA in the media. LNCaP cells treated with DHA + TXT increased the percentage of cells in G2M phase of cell cycle compared to control, DHA or TXT alone. This study did not assess any other marker of cell cycle, but investigate apoptosis and reported a depolarization/collapse in mitochondrial membrane potential (MMP), which are an early sign of apoptosis as well as inhibition of the NFκB pathway with the combination of DHA + TXT [26]. These results suggest that DHA treatments alone or in conjunction with chemotherapy target the cell cycle at the G2M phase in multiple cancer models.

### 3.6. G2M Phase Cellular Markers

In normal cell cycle progression, the transition from G2 to M phase is marked by an increase in Cyclin B and CDK1 expression as well as an increase in expression of mitotic genes including *CDC25C* and in the neoplastic cell cycle, CDK1 is thought to be necessary for tumorigenesis [40]. Protein expression of these three markers was found to be decreased in MDA-MB-231 breast cancer cells [24] and CDK1 expression was lower in pancreatic cells after DHA treatment [23]. The three studies that assessed DHA in combination with chemotherapy focused on enhanced chemo-sensitization with DHA treatment and did not assess G2M phase markers [25,26,27], although Shaikh et al. did assess changes in MMP and NFκB candidate genes [26].

### 3.7. Multi Cell Cycle Phase Analysis

A comparison of the response to DHA in cells harbouring a wild type p53 versus a mutated p53 was made in a colorectal cancer model. An effect on the cell cycle after DHA treatment occurred in G1 in p53-mutated WiDr cells and while an effect in G2M was seen in p53 + COLO205 cells. While COLO205 cells went on to programmed apoptotic death, WiDr cells were not stimulated by DHA to undergo apoptosis, but rather had reduced proliferation [28]. This suggests that the fate of the cell and the phase of the cell cycle in which it gets arrested in response to DHA treatment may be dependent on the p53 status of the cell. In synchronized KLP-1 breast cancer cells treated with 200 μmol/L DHA or 97 μmol/L conjugated DHA (CDHA, a geometric isomer of DHA prepared by alkaline isomerization); differential effects were seen in cell cycle response. After 24 h, in CDHA-treated cells, the percentage of cells in G1 increased by 33% compared to control, whereas in DHA-treated cells the percentage found in G2M increased by 22% compared to control [30]. This suggests that the formulation of DHA is also important in eliciting a cell cycle response. A comprehensive study of chemotherapy-resistant colorectal cancer SW620 cells reported that DHA treatment reduced the expression of many G1 and G2 genes both at transcript and protein level. In G1, *Cyclin D1*, *CDK1*, *CDK2* and *CDK4* had decreased expression (protein and mRNA) while p21 and 14-3-3 (stratifin) were decreased with 70 μM DHA treatment. These factors are consistent with arrest at G1. Up-regulation of stratifin is an important event in cell cycle arrest as it anchors CDK1 in the cytoplasm and from there it is unable to form a complex with Cyclin B1 and induce mitosis [29].

In G2M, there was a 2.5-fold increase of cells corresponding with a down-regulation of mRNA in the following G2M checkpoint proteins: *AURKA*, *AURKB*, *BIRC5*, *BUB1*, *CCNA2*, *CCNA2*, *CCNB2*, *CNF*, *CDC2/CDK1*, *CDC20*, *CDC25B*, *CDC25C*, *CENPE*, *FOXO3A* and *PLK* and decreased protein expression in Cyclin B2, CDC25B and CDC25C [29]. These findings suggest that, in these cells after DHA treatment, p21 inhibits progression through the cell cycle resulting in arrest in either G1 or G2 depending on what phase of the cell cycle a cell is in upon treatment.

## 4. Conclusions and Future Directions

Treatment with DHA has been demonstrated in cell lines and preclinical models to inhibit cell proliferation or growth across a wide spectrum of cancers. There is considerable evidence that treatment with DHA is able to elicit arrest in the G1 phase, S phase and possibly G2M phase (particularly when co-treated with cytotoxic drugs) and decreases the expression of cyclins and other cell cycle markers throughout the cell cycle (Figure 1). The efficacy and the specificity of DHA likely depend on two main factors: (1) the molecular properties or type (invasiveness) of each cancer; and (2) the variability in the experimental conditions, including time, concentration and synchronization of cells.

Emerging evidence highlights the complexity of treating human cancer cells due to extensive mutations including p53 [41], KRAS [42], PIK3CA and PTEN [43], and indeed mutational status might contribute to the effect of DHA. While mutational status is known for many immortalized cancer cell lines [44], cellular response to DHA based on mutation has not been specifically addressed in these studies and there are insufficient data available at this time to systematically look at this as a possible contributor.

Half of the studies reviewed synchronized the cells and this could be a key factor affecting not only the time point during which cells arrest in the cell cycle, but also which cell cycle markers are involved. While serum starvation does correct for synchronization, it may also induce additional stress to cells and make them more vulnerable to DHA treatment, possibly adding other variables that confound the ability to compare between cell types and experiments.

The ability of DHA to induce an effect at specific points in the cell cycle could be in part due to the broad ranges of doses (from 10 to 300 μM) and methods of exposing cells to fatty acids (free fatty acids or bound to albumin) used in reviewed studies. This highlights the need for a more biological marker of exposure such as membrane *n*-3 incorporation which might help explain the variability in responses reported. It may also be helpful to adjust for the different modes of administering: bovine serum albumin (BSA)-conjugated versus free fatty acids as it is likely that free fatty acids are taken up more readily than BSA-conjugated fatty acids [45].

The current evidence presented suggests G1 arrest occurs after a longer incubation compared to a shorter incubation time for G2 arrest. This could be due to the fact that, while G2M arrested cells become apoptotic and subsequently enter programmed cell death, G1 cells do not undergo apoptosis, but it could also be attributed to the limited data available. There are substantial gaps in the literature. Of the 20 in vitro studies, only five specifically studied the effects of DHA on the cell cycle while the majority focused on apoptosis. The limited number of studies that have focused on cell cycle arrest and *n*-3 PUFA treatment results in the inability to clearly elucidate the mechanisms that are affected by DHA treatment. While the role of lipid peroxidation on DHA action and apoptosis has been well established [6,7], further studies are needed to provide evidence that changes in lipid peroxidation/oxidative stress may impact cell ability to proceed through the cell. Additionally, the effect of DHA on nuclear receptors [46] or transcription factors and subsequent implications on cell cycle progression will need to be explored. Furthermore, to really understand the effect of DHA on cell cycle arrest, future studies should include analysis of cell cycle markers throughout the entire cell cycle as only one study (with the chemotherapy resistant cells [29]) provided evidence of markers throughout the entire cell cycle. In addition, the activation status of cyclins and cyclin dependent kinases are influenced by not only their expression levels but also their phosphorylation state [10,47] and it will be necessary to investigate whether the effect of DHA is due to changes in protein level versus changes in phosphorylation. Furthermore, understanding how DHA interferes with cell cycle will be important to determine if there is a role for DHA treatment in combination with chemotherapy for cell cycle inhibition.

## Figures and Tables

**Table 1:** Overview of in vitro studies investigating cell cycle in cancer cells treated with docosahexaenoic acid.

Cell Cycle	Cancer Model	Cancer Cell Line	Treat	Cell Cycle Markers	Other Markers	Reference
G1	Leukemia	KG1A	150 μM	↑ cells in G1 and ↓ in G2M	↑ apoptosis; ↑ DNA fragmentation; NC BCL2, ↑ Bax expression	[9]
G1	Neuroblastoma	LA-N-1; (HEK-293; WRL-68-control)	0–70 μM	↑ in cells in G1; ↓ expression of CDK2 and Cyclin E	↑ apoptosis; ↑ PS extern.; ↓ MMP; ↓ BCL-XL and ↑ Bax, Casp-3 and -9; Casp 8 NC	[10]
G1	Colorectal	HT-29	150 μM	↑ in cells in G1; ↓ Cyclin D1, E and A activation; ↓ expression of Cyclin A and pRb; ↓ E2F-1 DNA binding activity	NA	[11]
G1	Breast	4T1 (Mouse); MCF-7 (Human)	25–100 μM	↑ cells in G1; ↓ β-catenin, c-myc, Cyclin D1 in 4T1 cells	↑ apoptotic in 4T1 and MCF-7 cells	[12]
G1	Breast	MDA-MB-231, MCF-7, SK-BR-3, HCC1806	80 μM	↑ cells in G1; ↓ in p21 in MCF-7 and SK-BR-3, ↑ in HCC1086, NC in MDA-MB-231; NC in p27 or Cyclin D1	↑ apoptosis	[13]
G1	Breast	FM3A (Mouse)	10 μM	↑ cells in G1; ↑ p27; ↓ MAPK expression; NC p27 mRNA; ↓ Cyclin E, pCDK2 expression; NC Cyclin D; ↓ pRB	NA	[14]
G1	Breast	MCF-7, ZR-75-1, SK-BR-3, MCF-10A	100 or 300 μM	↑ in cells in G1; ↑ in sub G1; ↑ p21 (mRNA and protein) in MCF-10A; NC in G1; ↓ sub G1; ↓ p21 (mRNA) in MCF-7; ↑ in G2M; ↑ in G1; ↓ p21 (mRNA) SKBR3	↓ p-ERK ½ and STAT3 in SKBR3 and MCF-7 cells; ↑ p-ERK ½, STAT3; ↑ p53 all cell lines	[15]
G1	Gastric	AGS	7.5–45 μg/mL DHA; 1.5625–50 μg/mL 5-FU	↑ cells in G1 with DHA or 5-FU alone; ↑ cells in G1 more in combination; ↓ in S-phase	↓ in METC I, II, V expression	[16]
S	Leukemia	E6-1	0–30 μM	↑ cells in S; ↓ Cdk2, pRb and Cyclin A expression; ↑ p21	4-fold ↑ ceramide formation; ↓ Casp-3 expression	[17]
S	Melanoma	SK-Mel-110 and SK-Mel-29 (control)	0.5–5 μg/mL	Two fold ↑ SK-Mel-110 cells in S; ↓ pRb in SK-Mel-110; NC Cyclin D, E, p21, p27	↑ apoptotis in SK-Mel-110	[18]
S	Liver	MHCC97L	0–200 μM	↑ in cells in sub G1; prolonged S phase; ↓ in Cyclin A, E and CDK2	↓ *COX-2* mRNA; NC protein expression; ↓ Hsp27, GRP78, N-myc protein; ↑ SOD2	[19]
G2M	Pancreatic	MIA PaCa-2	10–100 μM *n*-3 emulsion	↑ in cells in G2; ↓ in G1, 13% ↑ in S-phase; large sub G1; ↓ Cdc2 (Cdk1) expression	↑ in apoptotic cells; ↓ BCL-2 expression; ↑ PARP cleavage product	[20]
G2M	Breast	MDA-MB-231	30–100 μM DHA	↑ cells in G2M; ↓ CDK1, Cyclin B1, Cyclin A, CDC25C, Cyclin B1p-Ser126 and NC Cyclin E	↑ apoptotis with ↑ concentrations DHA	[21]
G2M	Leukemia	EHEB, JVM-2 and MEC-2	50 μM; 0.75 μM Dox	↑ in cells in G2M with DHA alone; ↑ in G2M with DHA + Dox (EHEB, JVM-2 and MEC-2); ↑ in G2/M DHA + vincristine (JVM-2 and EPA) ↑ in G2/M DHA + fludarabine (EHEB)	↑ cell death from Dox in EHEB, JVM-2 and MEC-2; ↑ cell death from vincristine in JVM-2 and MEC-2 and fludarabine in EHEB	[22]
G2M	Prostate	LNCaP, DU145, PC3	25 μM; 0.6 nM TXT	↑ sub G1 cells; no diff between DHA, TXT, and combo; ↑ in G2M in LNCaP cells; >DHA + TXT than other treatments alone	↑ MMP collapse in DHA + TXT; ↑ MAP2K4, TNFRSF11A, RIPK1; ↓ FADD, AKT1, MAX (microarray); RT-PCR opposite values	[23]
G2M	Colorectal	CaCo2	FO (10–50 uM EPA 2:1 EPA:DHA); 0.25–1.0 μmol/L 5-FU	↑ cells in G2M with FO, ↑ in S with 5-FU and ↑ cells in S and ↓ in G2M with 5-FU and FO combined	↑ in apoptotic cells	[24]
G1 and G2M	Colorectal	COLO205 (wt p53) and WiDr (mutated p53)	125 μM	↑ in G1 in WiDr; ↑ G2M in COLO205	↓ proliferation in WiDr (NC in COLO205), ↑ apoptosis in COLO205, NC in WiDr	[25]
G1 and G2M	Colorectal	SW620 (chemotherapy resistant)	70 μM	↓ Cyclin D1, D3, A2, B2, F, CDK1, CDK2, CDK4, PCNA, CDC25B, CDC25C; ↑ p21, 14-3-3; ↓ mRNA transcript: G1/S: *CCND1*, *CCND3*, *CCNG2*, *CDC42*, *CDC45L*, *CDC7*, *CDK2*, *CDK2AP*, *CDK4*, *CIP1/P21*, *CDKN1A*, *E2F1*, *PCNA*, *UNG*, *G2M: AURKA*, *AURKB*, *BIRC5*, *BUB1*, *CCNA2*, *CCNA2*, *CCNB2*, *CNF*, *CDC2/CDK1*, *CDC20*, *CDC25B*, *CDC25C*, *CENPE*, *FOXO3A*, *PLK1*; ↑ p21, 14-3-3 protein	↑ Gadd-45A, Gadd45B and Gadd34, Casp-4, 7, TNFRSF10B mRNA; ↓ NFκB, p38-P, α, β-livin, ↑ t-livin (protein); NC total p38 or Survivin (protein)	[26]
G1 and G2M	Breast	KLP-1	97 (CDHA) 270 (DHA) μmol/L	↑ cells in G2 with DHA; ↑ cells in G1 with CDHA; Cyclin D1; ↑ p21 expression	↑ apoptosis; ↑ p53; ↓ BCL-2; NC Bax	[27]
G1 and G2M	Breast	MDA-MB-231 MCF-7	0–100 nmol/L Dox	↑ cells in G1 and G2M in MCF-7; ↑ G2M in MDA-MB-231; ↓ expression SKP2, p21, p27, Cyclin B, p53 in MCF-7; ↑ protein expression SKP2, Cyclin B, p53 and ↓ p21 MDA-MB-231	NA	[28]

CDHA, conjugated DHA; DHA, docosahexaenoic acid; Dox, doxorubicin; EPA, eicosapentaenoic acid; 5-FU, 5-fluorouracil; FO, Fish oil; NC, no change; METC, mitochondrial electron transfer chain; MMP, mitochondrial membrane potential; NA, not applicable; p21, also known as p21^Waf1^; p27, also known as p27^KIP1^; TXT, docetaxel; wt, wild type.

**Table 2:** Overview of in vivo studies investigating cell cycle in cancer cells treated with docosahexaenoic acid.

Animal Model	Tumour Model	Treatment/Diet	Results	Reference
BALB/c mice	KLP-1	0, 0.2%, 1.0% CDHA	NC body weight; ↓ in tumour volume and ↓ in metastases in 1.0% CDHA, but NC in tumour weight	[27]
Rats	mammary tumours induced with 1M1N	high *n*-3 diet (3:1 EPA:DHA, 45 g/kg diet)	↓ in Cyclin D1, pRB ↑ p21, ↑ p27 protein expression; ↑ apoptotic markers	[29]
BALB/c mice	4T1; mammary fat pad	5% fish oil	↓ tumour weight; ↓ in Cyclin D1, c-myc, B-catenin ↑ TUNEL + cells	[12]

CDHA, conjugated DHA; DHA, docosahexaenoic acid; EPA, eicosapentaenoic acid; 1M1N, 1-methyl-1-nitrosurea; NC, No change; TXT, docetaxel.

## Abbreviations

5-FU	5-Fluorouracil
AA	Arachidonic acid
ALA	Alpha linolenic acid
AURK	Aurora kinase
BHT	Butylated hydroxyanisole
BSA	Bovine serum albumin
CDHA	Conjugated DHA
CDK	Cyclin dependent kinase
DHA	Docosahexaenoic acid
DOX	Doxorubicin
ER	Estrogen-receptor
EPA	Eicosapentaenoic acid
FO	Fish oil
G1	Gap 1
G2	Gap 2
HER2	Human epidermal growth factor receptor 2/erbB-2 receptor tyrosine-protein kinase ERBB2
LA	Linoleic acid
LCPUFA	Long chain polyunsaturated fatty acid
METC	Mitochondrial electron transfer chain
MMP	Mitochondrial membrane potential
mRNA	Messenger RNA
PLK1	Polo-like kinase 1/Serine/threonine-protein kinase
PPAR	Peroxisome proliferator-activated receptors
PR	Progesterone-receptor
pRB	Retinoblastoma protein
RTPCR	Real time quantitative PCR
TXT	Docetaxel
